# *DeltaNp63alpha-*Mediated Induction of Epidermal Growth Factor Receptor Promotes Pancreatic Cancer Cell Growth and Chemoresistance

**DOI:** 10.1371/journal.pone.0026815

**Published:** 2011-10-28

**Authors:** Alexey V. Danilov, Divas Neupane, Archana Sidalaghatta Nagaraja, Elena V. Feofanova, Leigh Ann Humphries, James DiRenzo, Murray Korc

**Affiliations:** 1 Department of Medicine, Dartmouth-Hitchcock Medical Center, Lebanon, New Hampshire, United States of America; 2 Norris Cotton Cancer Center, Dartmouth-Hitchcock Medical Center, Lebanon, New Hampshire, United States of America; 3 Department of Pharmacology and Toxicology, Dartmouth Medical School, Hanover, New Hampshire, United States of America; University of Chicago, United States of America

## Abstract

Pancreatic ductal adenocarcinoma (PDAC) is highly resistant to current chemotherapy regimens, in part due to alterations in the p53 tumor suppressor pathway. p53 homolog p63 is a transcription factor essential for the development and differentiation of epithelial surfaces. However its function in cancer is controversial and its role in PDAC is not known. We discovered that ΔNp63α was the predominantly expressed p63 variant in pancreatic cancer cell lines. ΔNp63α protein and mRNA levels were high in T3M4, BxPC3 and COLO-357 pancreatic cancer cells and low in ASPC-1 and PANC-1 cells. Overexpression of ΔNp63α in PANC-1 cells and shRNA-mediated knockdown in T3M4 cells indicated that ΔNp63α promoted anchorage-dependent and -independent growth, motility and invasion, and enhanced resistance to cisplatin-induced apoptosis. Epidermal growth factor receptor (EGFR) signaling pathways contribute to the biological aggressiveness of PDAC, and we found that the motogenic effects of ΔNp63α were augmented in presence of EGF. Ectopic expression of ΔNp63α resulted in upregulation of EGFR and β1-integrin in PANC-1 cells. Conversely, ΔNp63α knockdown had an opposite effect in T3M4 cells. ΔNp63α potentiated EGF-mediated activation of ERK, Akt and JNK signaling. Chromatin immunoprecipitation and functional reporter assays demonstrated that ΔNp63α activated EGFR transcription. 14-3-3σ transcription was also positively regulated by ΔNp63α and we have previously shown that 14-3-3σ contributes to chemoresistance in pancreatic cancer cell lines. Conversely, shRNA-mediated knockdown of 14-3-3σ led to abrogation of the ΔNp63α effects on cell proliferation and invasion. Thus, p53 homolog ΔNp63α enhances the oncogenic potential of pancreatic cancer cells through trans-activation of EGFR and 14-3-3σ.

## Introduction

Studies in human disease demonstrate that most established tumors carry more than one genetic defect. Pancreatic ductal adenocarcinoma (PDAC) results from the successive accumulation of gene mutations [1]. Activating mutations in K-Ras oncogene and inactivation of tumor suppressors CDKN2A, p53 and SMAD4 are implicated in PDAC development and progression [2]. Genetically engineered mouse models have supported the “double-hit” hypothesis where introduction of either mutant p53 allele or biallelic deletion of ink4a/Arf in mice results in progression of pancreatic intraepithelial neoplastic lesions with local invasion and metastases [3], [4]. p53 mutations are found in 60 to 70% of PDAC. By contrast, p63, an ancestral member of p53 family, is rarely mutated in cancer.

Six variants of p63 are generated through transcription from two distinct promoters and alternative splicing. Isoforms transcribed from P1 contain a full-length trans-activation domain (TAp63α, β and γ). Transcription from P2 generates amino-terminally truncated variants (ΔNp63α, β and γ), whose exact role in cancer is not clear. The ΔNp63 variant is overexpressed in a variety of human cancers, including tumors of squamous cell origin (head and neck, lung), breast and bladder [5]. In head and neck squamous cell carcinoma and “triple-negative” breast cancer cells, ΔNp63 suppresses p73-dependent apoptosis and thus promotes tumor survival [6], [7]. By contrast, downregulation of ΔNp63α in urothelial carcinoma cell lines promotes cancer invasiveness [8], suggesting that the ΔN variant may function in a cell type-specific manner.

The role of p63 in PDAC is poorly understood. Here we demonstrate that the ΔNp63α variant is expressed at variable levels in PDAC cell lines, and provide evidence that ΔNp63α promotes pancreatic cancer cell growth, migration, invasion and chemoresistance. Via direct transcriptional activation, ΔNp63α leads to the up-regulation of epidermal growth factor receptor (EGFR) and 14-3-3σ, sensitizing cancer cells to EGF and enhancing their oncogenic potential.

## Methods

### Cell lines

Human pancreatic cancer cell lines ASPC-1, BxPC3 and PANC-1; HEK293 human embryonic kidney cells and H1299 human lung carcinoma cells were purchased from American Type Culture Collection (Manassas, VA). COLO-357 and T3M4 human pancreatic cancer cell lines were a gift from Dr.R.Metzgar (Duke University, Durham, NC). Cell lines were grown in RPMI or DMEM (HEK293) supplemented with 10% fetal bovine serum, 100 U/ml penicillin, and 100 µg/ml streptomycin (complete medium).

### Plasmid constructs

The human and mouse ΔNp63α expression plasmids and (p53RE)_5_-tk-luciferase plasmid were reported previously [9]. TAp63α expression plasmid was purchased from Open Biosystems (Huntsville, AL). pBV-14-3-3σ promoter BDS 2 3× (p53 binding site)-luc plasmid was obtained from Addgene (Cambridge, MA). pGL3-EGFR promoter (p53 binding sites)-luc plasmid was a gift from Dr. L. Pirisi [10]. Sequence modifications within a coding of a DNA-binding domain of the human ΔNp63α expression plasmid and within EGFR promoter p53 binding site 1 were introduced using the Quick-Change Site Directed Mutagenesis Kit (Stratagene, La Jolla, CA).

### Immunoblotting

Cells were lysed in RIPA buffer (20 mM Tris, 150 mM NaCl, 1% NP-40, 1 mM NaF, 1 mM Sodium phosphate, 1 mM NaVO3, 1 nM EDTA, 1 nM EGTA, supplemented with protease inhibitor cocktail (Roche, Indianapolis, IN) and 1 mM PMSF). Proteins were analyzed by immunoblotting as previously described [11]. The following antibodies were used: p63 (4A4; Millipore, Billerica, MA; 1∶500); TAp63γ (Li et al., 2006; 1∶1,000), 14-3-3σ (Abcam, Cambridge, MA; 1∶500), ERK-2 (C-14; Santa Cruz Biotechnology, Santa Cruz, CA; 1∶10,000), p53 (DO-1; Santa Cruz Biotechnology; 0.4 µg/mL), EGFR (Calbiochem; 1∶2,000), cleaved poly(ADP-ribose) polymerase (PARP), cleaved caspase-3, ERK-1/2, phospho-ERK1/2 [T202/Y204], Akt, phospho-Akt [S473] (Cell Signaling Technology, Danvers, MA; 1∶1,000), active JNK (Promega, Madison, WI), horseradish peroxidase-conjugated anti-mouse and anti-rabbit antibodies (BioRad; 1∶5,000).

### Reverse transcription polymerase chain reaction (RT-PCR)

Total RNA was isolated using RNeasy Mini Kit (Qiagen, Valencia, CA). cDNA was synthesized from 1 µg RNA by random hexamer priming using the Superscript III RT Kit (Invitrogen, Carlsbad, CA). Semi-quantitative RT-PCR was carried out using forward and reverse primers specific for p63 isoforms as previously published [12]. The following PCR cycling conditions were used: 94°C for 3 min, 35 cycles of 94°C for 40 sec, 55°C for 40 sec, and 72°C for 1.5 min; and 72°C for 4 min.

For the analysis of ΔNp63 and TAp63 transcript expression quantitative real time PCR (Q-PCR) was performed in a 7300 Sequence Detector using Universal PCR Master Mix according to manufacturer's instructions (Applied Biosystems, Foster City, CA), template cDNA and gene specific probes (Hs00978339_m1 [ΔNp63]; Hs00978349_m1 [TAp63]; Applied Biosystems). All samples were analyzed in duplicates.

### Transient transfections, adenoviral infection and luciferase assays

PANC-1 and T3M4 cells were transiently transfected with ExGen 500 *in vitro* Transfection Reagent (Fermentas Life Sciences, Glen Burnie, MD), using equal amounts of experimental or control plasmid. Cells were incubated for 48 hours and protein expression was verified by immunoblotting. The control and ΔNp63α-expressing adenovirus were used as described [13]. ASPC-1 and PANC-1 cells were infected with adenovirus at MOI of ∼5 in complete medium for 24 hours.

For luciferase assays, PANC-1 cells were co-transfected with experimental plasmid or control and the luciferase reporter construct (pBV-14-3-3σ promoter BDS 2 3× (p53 binding site)-luc; or pGL3-EGFR promoter-luc) along with pCMVβ vector (Clontech Laboratories, Mountain View, CA). The amount of DNA per transfection was kept constant by using empty pcDNA3.1 vector. Cells were harvested 48 hours post-transfection and luciferase assays were performed using the Dual-Luciferase Reporter Assay System (Promega). Relative light units were determined using a luminometer (LMaxII^384^, Molecular Devices, Sunnyvale, CA) for firefly luciferase. β-galactosidase activity was determined using a colorimetric method to normalize transfection efficiency [14].

### Lentiviral shRNA mediated gene silencing

Lentivirus containing shRNA targeting α-specific, TAp63 specific, all p63 isoforms and sh control were described previously [15]. Lentivirus-based 14-3-3σ-targeting and control shRNA were also described by us previously [11]. Lentiviral particles were produced by four plasmid transfection system [11]. Knockdown of p63 isoforms and 14-3-3σ in T3M4 and PANC-1 cells was confirmed by Western blotting and Q-PCR.

### 3-(4,5-Dimethylthiazol-2-yl)-2,5-diphenyltetrazolium bromide (MTT) assay

Cells were plated in 96-well plates (3000 cell per well, 6 wells per sample) and cultured in the absence or presence of 10 µg/mL cisplatin (Sigma-Aldrich, St. Louis, MO) for 48 hours. MTT (Sigma-Aldrich) was added at a final concentration 0.55 µg/mL. After additional 4 hours incubation, absorbance at 570 nm was determined using an Emax precision microplate reader (Molecular Devices). We have previously observed that in pancreatic cancer cell lines the MTT assay correlates with cell growth as determined in a doubling and [^3^H]thymidine incorporation assays [16].

### Soft agar assays

Soft agar assays were set up in twelve-well plates, each well containing a bottom layer of 0.5% Difco agar noble (BD Biosciences), a middle layer of 0.3% agar including 1500 cells, and a top layer of 0.3% agar. The plates were incubated for 14 days. Next, 150 µL of 5 mg/mL MTT solution were added to each well. After incubation at 37°C for 4 hours plates were photographed and colonies were counted.

### Cell migration and invasion assays

Cell motility was assessed in *in vitro* wound healing and Transwell migration assays. For wound healing assays, cells were grown to confluency in 6-well tissue culture plates, and serum-starved for 12 hours. The resulting cell monolayer was scratched with a 10 µL pipette tip generating two parallel wounds and incubated for 18 hours in serum-free medium (SF; 0.1% bovine serum albumin) in the absence or presence 1 nM EGF (Millipore). Photographs were taken of each well at four marked locations under 40× magnification at zero and 18 hours after wounding. The wound area of matched pictures was measured using the ImageJ software (National Institutes of Health). For Transwell migration assays, cell were suspended in 100 µL SF medium and placed onto the upper compartment of Transwell chambers (8 µm pore size, Corning Incorporated, Corning, NY). For invasion assays, cells were suspended in 500 µL SF medium and placed onto upper compartment of Matrigel-coated Transwell chambers (8 µm pore size, BioCoat Matrigel Invasion Chambers; BD Biosciences; Franklin Lakes, NJ). In both Transwell assays, the lower compartment was filled with 750 µL SF medium in the absence or presence of 1 nM EGF. After 18–20 h, membranes were fixed in methanol, stained with toluidine blue (Fisher Scientific, Pittsburgh, PA) and counted using a light microscope.

### Formaldehyde crosslinking, chromatin immunoprecipitation (ChIP) and PCR amplification

For DNA-histone crosslinking, 2×10^6^ cells were incubated with 1% formaldehyde in complete medium for 10 minutes. Cells were washed twice in ice-cold PBS and lysed in SDS Lysis Buffer (Millipore) complete with protease inhibitor cocktail (Roche). Protein lysates were sonicated to yield chromatin fragments of ∼500 bp as assessed by agarose gel electrophoresis. After pre-clearing, protein lysates were incubated at 4°C overnight with 2 µg p63α antibodies (H-129, Santa Cruz) or with rabbit IgG as isotype-specific control antibodies (Santa Cruz). Immunocomplexes were washed and DNA was purified using the ChIP Assay Kit (Millipore) and QiaQuick PCR Purification Kit (Qiagen) according to the manufacturer's instructions. PCR conditions and primers for 14-3-3σ consensus binding sites 1 and 2 were previously reported [17]. The following PCR primers and conditions were used for the EGFR promoter p53 binding site: forward primer 5′ GGCCGCTGGCCTTGGGTC 3′; reverse primer 5′ GCCGTGCGCGGTGGTTG 3′; 1 cycle of 94°C for 5 min, 43 cycles of 95°C for 30 sec, 55°C for 30 sec, 72°C for 50 sec, followed by 1 cycle of 72°C for 10 min. PCR was performed using HotStarTaq polymerase Kit (Qiagen), with use of Q-Solution in the EGFR PCR assay.

### Cisplatin-induced apoptosis

Pancreatic cancer cells were incubated for 24 h in complete medium in the absence or presence of 5 or 10 µg/mL cisplatin for the initial 2 h, 6 h or the entire 24 h. Both floating and adherent cells were collected and subjected to immunoblotting.

## Results

### ΔNp63α expression in pancreatic cancer cell lines

**Figure 1:**
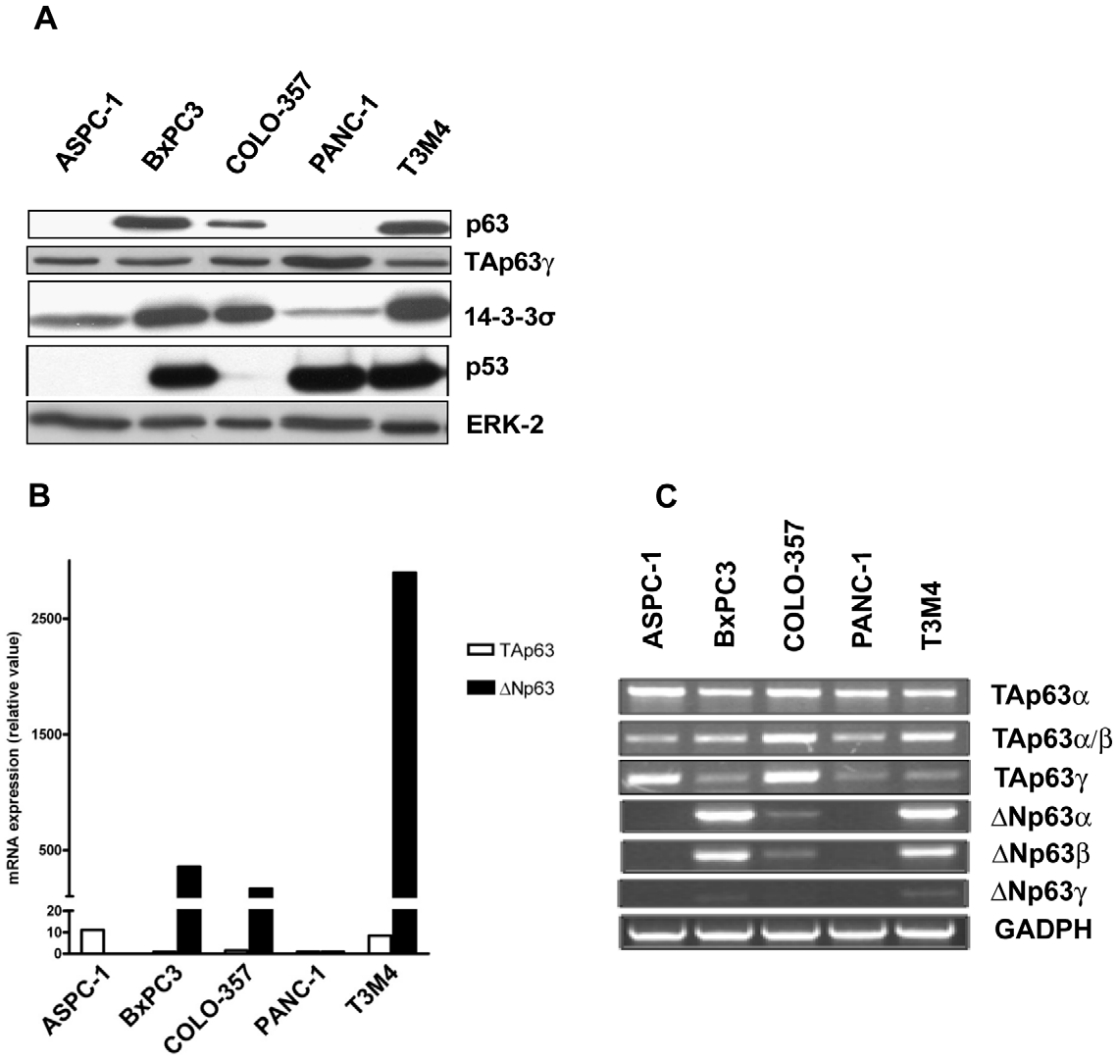
Expression of 14-3-3σ, p63 isoforms and p53 in pancreatic cancer cell lines. *A*, Protein levels of p63, 14-3-3σ, TAp63γ and p53 in pancreatic cancer cell lines. *B*, mRNA expression of TAp63 and ΔNp63 isoforms of p63 in pancreatic cancer cell lines. Total RNA isolated from PDAC cell lines was reverse-transcribed and subjected to real-time PCR with probes specific for ΔN and TA isoforms of p63. Results were normalized to 18S levels, and expressed as mean of two independent experiments done in duplicates in which similar results were obtained. *C*, mRNA expression of p63 splicing variants in pancreatic cancer cell lines. Levels were normalized to glyceraldehyde-3-phosphate dehydrogenase (GAPDH) values.

We found that p63 was differentially expressed in pancreatic cancer cell lines. p63 protein levels were highest in T3M4 and BxPC3 cells, intermediate in COLO-357 and below the detection level in ASPC-1 and PANC-1 cells (Fig. 1A). Since the p63 antibody (clone 4A4) recognized all six known isoforms of p63, we compared p63 mRNA transcript levels in the above cell lines. All the cell lines expressed low levels of TAp63 mRNA (Fig. 1B). By contrast, ΔNp63 mRNA transcripts were relatively elevated in BxPC3, COLO-357, and T3M4 cells (Fig. 1B).

To further elucidate whether a particular splicing variant of p63 is expressed in pancreatic cancer cell lines, we employed semi-quantitative RT-PCR with p63 splicing variant-specific primers (Table S1). We confirmed that TAp63α mRNA transcripts were expressed at relatively low and similar levels in all five cell lines. By contrast, ΔNp63α mRNA transcripts were only expressed in BxPC3, COLO-357, and T3M4 cells, whereas ΔNp63γ mRNA was barely detectable in BxPC3 and T3M4 cells (Fig. 1C). Although p63α can be detected in a specific PCR assay, isolated detection of p63β mRNA transcripts is unreliable since its C-terminus is not unique (Fig. S1). In our experiments, expression of ΔNp63β mRNA transcript correlated with ΔNp63α expression, probably due to non-specific amplification of ΔNp63α. Since we detected p63 protein migration at ∼68 kDa, and ΔNp63β protein migrates at ∼52 kDa [18]–[20], we concluded that ΔNp63α was the predominantly expressed p63 variant in pancreatic cancer cells.

### Oncogenic effects of ΔNp63α in pancreatic cancer cells

**Figure 2:**
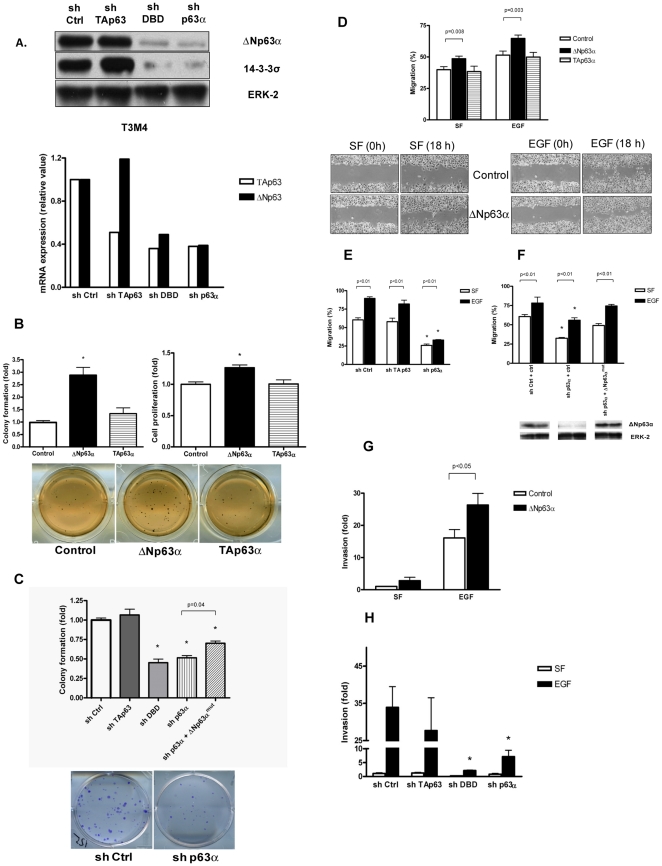
Effect of ΔNp63α on anchorage-independent growth, motility, and invasion in pancreatic cancer cells. *A*, T3M4 cells were infected with GFP-expressing virus, or p63-specific shRNA complementary to DBD, TA and α-specific domains of p63. Whole-cell protein lysates were subjected to immunoblotting (top panel). Total RNA was isolated, reverse-transcribed and subjected to real-time PCR with probes specific for ΔN and TA isoforms of p63. Results were normalized to 18S levels (bottom panel). Knockdown of p63 isoforms was routinely monitored during the subsequent experiments. *B*, ΔNp63α stimulates anchorage-independent growth of PANC-1 cells in soft agar assay (left and bottom panels) and cell proliferation in MTT assay (right panel). PANC-1 cells were transiently transfected with ΔNp63α-expressing vector, TAp63α or control vector. Cell were plated in soft agar at a density of 1500/well of a 12-well plate, four wells per sample. Colonies were counted after 14 days of incubation. For MTT assay cells were plated in 96-well plates, six wells per sample. MTT was added after incubation for 48 h. Data are the mean ± SE of four independent experiments. *, p<0.001 compared with control. *C*, Downregulation of ΔNp63α slows proliferation of T3M4 cells in a clonogenic assay. Reconstitution of ΔNp63α partially restores the proliferative ability. Cells were plated on 6 well plates at a density of 500 cells/well. 14 days later, plates were fixed in 3∶1 methanol:glacial acetic acid and stained with 2% crystal violet. Data are the mean ± SE of three independent experiments done in triplicates. *, p<0.01 compared with control (sh ctrl). *D–F*, Effect of p63 on cell motility measured in wound-healing assays in PANC-1 (*D*) and T3M4 cells (*E, F*). Cells were incubated in serum-free (SF) conditions in the absence or presence of 1 nM EGF for 18 h after making a scratch. Quantitative analysis of the images was performed. Ectopic expression of mouse ΔNp63α in sh p63α T3M4 cells resulted in a partial restoration of cell motility (*F*); corresponding ΔNp63α protein levels shown below. Data are the m ± SE of at least three independent experiments. Representative pictures shown (magnification ×40). *, p<0.001 compared with control (sh ctrl). *G and H*, Effect of ΔNp63α on the invasion in Matrigel chambers. PANC-1 (*G*) or T3M4 (*H*) cells were plated in Matrigel chambers (5×10^4^/ml) and incubated in absence (SF) or presence of 1 nM EGF for 18 hours. Effect was normalized to invasion of control in SF conditions. *, p<0.001 compared with control (sh ctrl).

To study the biologic effects of ΔNp63α in PDAC, we manipulated its expression in PANC-1 and T3M4 cells, which had low and high endogenous levels of ΔNp63α, respectively (Fig. 1). PANC-1 cells were transiently transfected with a ΔNp63α-expressing vector (PANC-1ΔN), a TAp63α-expressing vector (PANC-1TA), or control plasmid. Lentiviral-mediated shRNA was used to down-regulate p63 gene isoforms in T3M4 cells. Two different shRNA sequences, one complementary to a sequence within a p63 DNA-binding domain (sh DBD) and thus targeting all isoforms of p63, and another complementary to a sequence within sterile alpha motif domain (SAM) and thus targeting TAp63α and ΔNp63α (sh p63α) were determined to reduce the ΔNp63α protein and transcript levels in T3M4 cells (Fig. 2A). While shRNA targeting trans-activation domain (sh TAp63) resulted in reduced TAp63 transcript levels, this did not translate into an appreciable change in the total p63 protein levels (Fig. 2A), confirming our observation that ΔNp63α variant predominates in pancreatic cancer cell lines.

Since anchorage-independent growth is a hallmark of malignant cells, we studied whether ΔNp63α has an effect on anchorage-independent growth and cell proliferation in PDAC. PANC-1ΔN, but not PANC-1TA cells exhibited increased colony formation in soft agar assays and increased cell proliferation in MTT assays (Fig. 2B). Since T3M4 cells are incapable of forming colonies in soft agar, proliferation was studied in a clonogenic assay. As compared with control cells, proliferation of T3M4 cells was reduced in response to shRNAs that target the DBD or the α-specific C-terminus and unchanged in response to sh TAp63 (Fig. 2C). Transient overexpression of mouse ΔNp63α cDNA, which carries a silent mutation in the region targeted by the α-specific shRNA, resulted in a partial rescue of the proliferative ability of sh p63α T3M4 cells, as compared with cells transfected with control vector (Fig. 2C).

ΔNp63α is known to regulate the adhesion program in mammary epithelial cells and keratinocytes [15]. Given the importance of cell adhesion in tumor invasion and progression, we studied the effects of ΔNp63α on the motility and invasive capabilities of the pancreatic cancer cells. In wound-healing assays, PANC-1ΔN cells demonstrated enhanced motility in SF conditions, while overexpression of TAp63α had no effect (Fig. 2D). Since activation of the EGFR pathway is associated with increased tumor aggressiveness and decreased survival in PDAC [21], we sought to determine the effect of EGF on motility. EGF stimulation enhanced migration in PANC-1ΔN and control cells (Fig. 2D). Similarly, T3M4 cells expressing sh p63α, but not sh TAp63, exhibited reduced motility both at baseline and in presence of EGF (Fig. 2E). Transient overexpression of mouse ΔNp63α in T3M4 cells expressing sh p63α resulted in a partial rescue of the motility phenotype (Fig. 2F). Manipulation of ΔNp63α expression levels had no effect on the invasion of pancreatic cancer cells in Matrigel chambers in SF conditions (Fig. 2G, H). However, stimulation with EGF led to a dramatic increase in the invasive capability of PANC1-ΔN cells (Fig. 2G). Consistent with the above findings, shRNA-mediated suppression of ΔNp63α, but not TAp63, significantly reduced the ability of T3M4 cells to invade through Matrigel in response to EGF stimulation (Fig. 2H).

Collectively, our results indicate that ΔNp63α enhances the colony-forming, proliferative and invasive capacity of the pancreatic cancer cells.

### ΔNp63α upregulates EGFR and sensitizes pancreatic cancer cells to the effects of EGF

**Figure 3:**
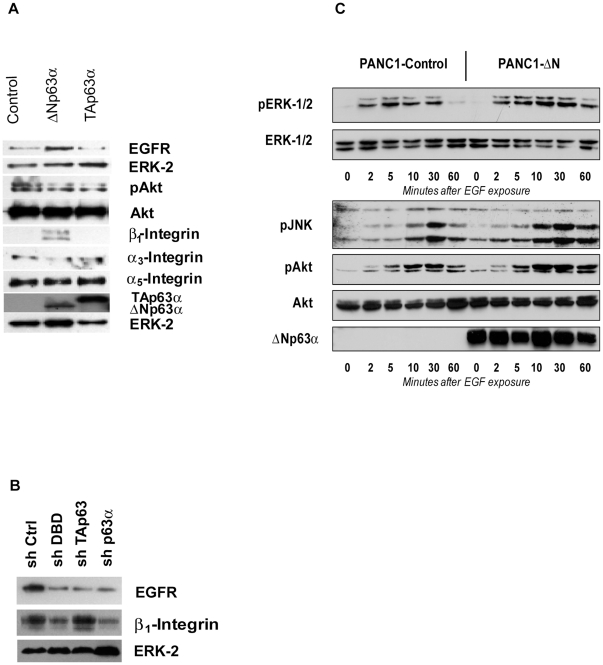
ΔNp63α upregulates EGFR expression and signaling in pancreatic cancer cells. *A*, Ectopic expression of ΔNp63α results in increased expression of EGFR and β1-integrin in PANC-1 cells. After cells were incubated for 48 hours, total protein lysates were subjected to immunoblotting. A representative image of three independent experiments is shown. *B*, Downregulation of ΔNp63α in T3M4 cells results in decreased expression of EGFR and β1-integrin. A representative image of three independent experiments is shown. *C*, PANC-1ΔN and PANC-1 control cells were stimulated with EGF during indicated time periods. Protein lysates were subjected to immunoblotting. A representative image of three independent experiments is shown.

Compared with the normal pancreas, EGFR protein and mRNA are expressed at high levels in pancreatic cancer [22]. EGFR mRNA expression predicts decreased survival and poor response to chemotherapy in patients with PDAC [23]. In our work, ΔNp63α dramatically potentiated pancreatic cancer cell motility and invasive abilities in the presence of EGF. To explain this phenomenon, we studied the effect of ΔNp63α on EGFR levels. We determined that engineered expression of ΔNp63α in PANC-1 cells enhanced EGFR expression, while ectopic TAp63α had no effect (Fig. 3A). Consistent with this observation, we found that EGFR levels were decreased in T3M4 cells in response to shRNA-mediated suppression of ΔNp63α (Fig. 3B), thus documenting a direct correlation between ΔNp63α and EGFR expression in pancreatic cancer cell lines. To study the effects of ΔNp63α overexpression on EGFR signaling, we assessed activation of downstream kinases upon EGF stimulation. Treatment of PANC1-ΔN cells with EGF resulted in enhanced phosphorylation of ERK, Akt and JNK (Fig. 3C). In those cells, all three kinases exhibited increased level of activation at as early as 10 minutes after cell stimulation, and ERK activity persisted for 60 minutes.

Next, we employed a tyrosine kinase inhibitor to assert that the effects of ΔNp63α were mediated through the EGFR pathway. Erlotinib is a reversible inhibitor of the EGF signaling, which associates with the ATP-binding site of the receptor. Incubation of PANC-1 cells with 1 µM erlotinib attenuated the ΔNp63α-mediated enhancement of colony formation, motility and invasion (Fig. S2), thus excluding a possibility of a non-specific effect. By contrast, p63 protein levels were not affected when cells were incubated with either EGF or erlotinib (data not shown).

Previous reports suggested that ΔNp63α is able to regulate cell adhesion proteins in mammary epithelial cells [15]. Since integrin-mediated tumor cell interactions with the extracellular matrix play a critical role in determining the invasive phenotype in PDAC, we investigated whether ΔNp63α had an effect on integrin expression in pancreatic cancer cells. Ectopic expression of ΔNp63α in PANC-1 cells increased expression of β1-integrin, whereas the expression of α3- and α5-integrins was unchanged (Fig. 3A). Conversely, shRNA-mediated suppression of ΔNp63α led to a decrease in β1-integrin in T3M4 cells (Fig. 3B).

Thus, ΔNp63α contributes to the oncogenic potential of pancreatic cancer cells, at least in part, through upregulation of EGFR and β1-integrin.

### ΔNp63α contributes to chemotherapy resistance in pancreatic cancer cells

**Figure 4:**
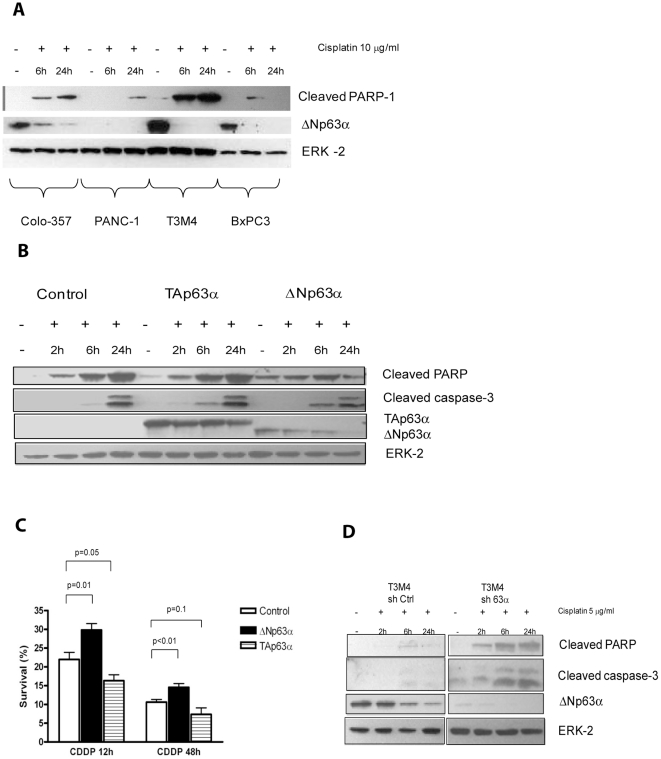
ΔNp63α contributes to chemoresistance in pancreatic cancer cells. *A*, Effect of cisplatin on ΔNp63α in PDAC. Cells were incubated with 10 µg/mL cisplatin for the initial 6 or full 24 hours. Apoptosis was assessed by monitoring PARP cleavage. *B–D*, Effect of ΔNp63α on cisplatin-induced apoptosis in PANC-1 (*B, C*) and T3M4 cells (*D*). Cells were incubated with 5 µg/mL of cisplatin during indicated time periods. Cells were pelleted after 24 h of incubation, proteins isolated and run on SDS-PAGE gel. Apoptosis was assessed by monitoring PARP and caspase-3 cleavage. A representative image of at least three independent experiments is shown. PANC-1 cells were incubated with 10 µg/mL of cisplatin for the first 12 hours or full 48 hours. MTT assay was done as described in materials and methods. Data are the mean ± SE of three independent experiments.

Since PDAC is notoriously resistant to chemotherapy agents, we studied the role of ΔNp63α in chemotherapy resistance. It has been previously shown that cisplatin induces degradation of ΔNp63α via stimulation of IκB kinase [24]. Consistent with those data, incubation of pancreatic cancer cell lines with 10 µg/ml of cisplatin resulted in degradation of endogenous ΔNp63α. This effect of cisplatin was evident after 6 hours of incubation and was accompanied by increased apoptosis in all tested cell lines (Fig. 4A). We then investigated whether ectopic expression of ΔNp63α would contribute to resistance to cisplatin-induced apoptosis in PDAC. PANC-1 cells were incubated for 24 hours in complete medium supplemented with 5 µg/mL cisplatin for 2, 6, or 24 hours. PANC-1ΔN cells exhibited a mild attenuation of apoptosis compared with control cells, as manifested by decreased PARP and caspase cleavage (Fig. 4B). This decrease in cisplatin-induced apoptosis correlated with an increase in viability of PANC-1ΔN cells as determined in an MTT assay, while PANC-1TA cells exhibited a slightly reduced survival (Fig. 4C). By contrast, T3M4 cells expressing sh p63α were sensitized to cisplatin-induced apoptosis, as demonstrated by an increased cleavage of PARP and caspase (Fig. 4D). Thus, ΔNp63α attenuates the sensitivity of pancreatic cancer cells to cisplatin.

### ΔNp63α upregulates 14-3-3σ in pancreatic cancer cell lines

Next we studied the mechanisms of ΔNp63α-mediated chemoresistance in PDAC. We have previously shown that 14-3-3σ dramatically enhances resistance to cisplatin-induced apoptosis in pancreatic cancer cell lines [11]. Westfall et al. demonstrated that ΔNp63α acts as a transcriptional repressor of the 14-3-3σ promoter in human epidermal keratinocytes [25]. By contrast, we observed that expression of ΔNp63α correlated with 14-3-3σ protein expression in pancreatic cancer cells (Fig. 1A). Additionally, silencing ΔNp63α was accompanied by reduction in 14-3-3σ protein level in T3M4 cells (Fig. 2A). 14-3-3σ is a p53 transcriptional target, yet BxPC3, PANC-1 and T3M4 cells are known to carry mutant p53 whose transcriptional activity is defective [26], while COLO-357 cells express low p53 protein levels (Fig. 1A). Hence, 14-3-3σ expression is unlikely to be dependent on p53 in PDAC.

**Figure 5:**
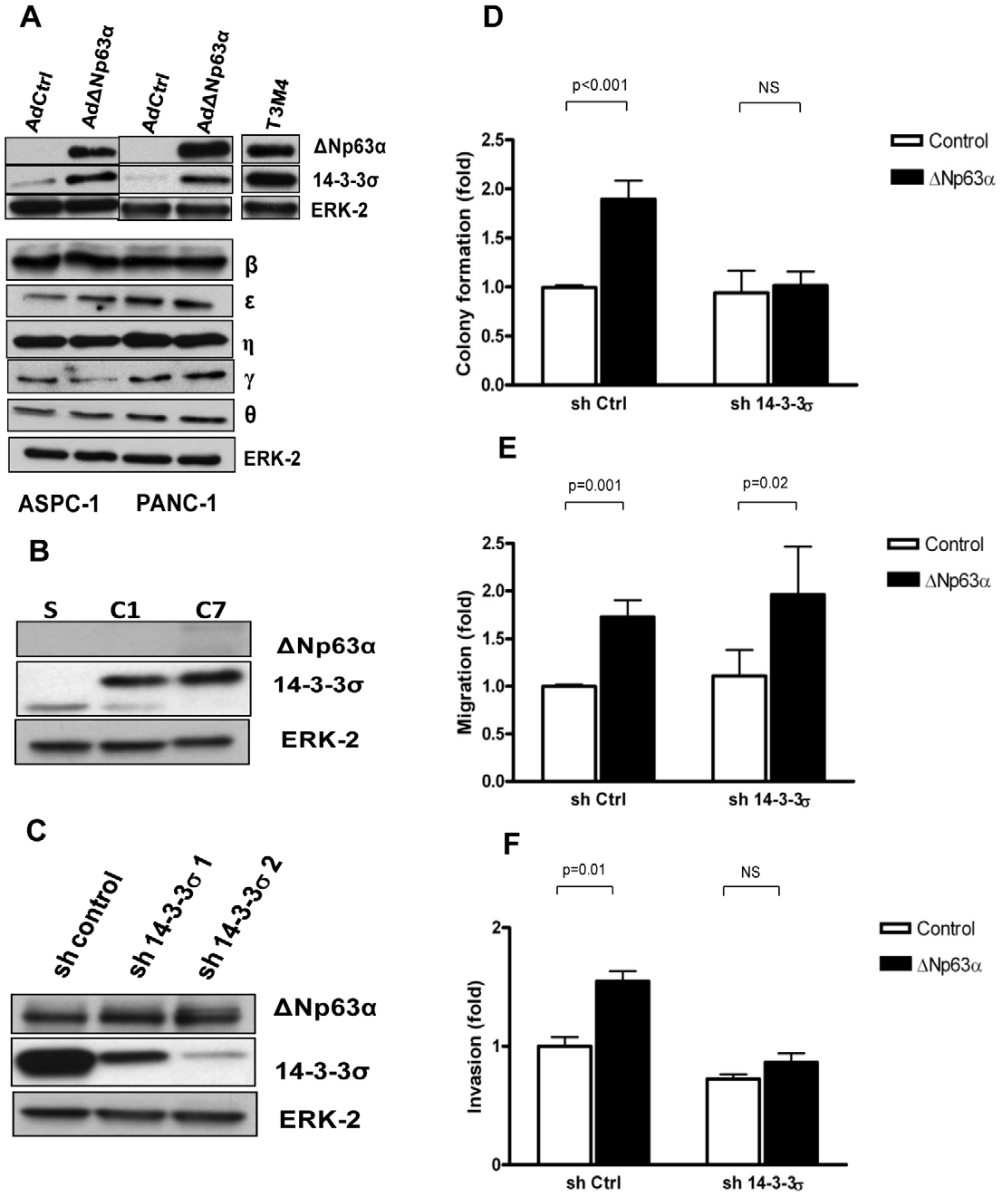
ΔNp63α upregulates 14-3-3σ in pancreatic cancer cells. A, ASPC-1 and PANC-1 cells were infected with adenovirus containing either empty vector or full length ΔNp63α, and whole cell protein lysate was prepared 48 hours after infection and probed with anti-ΔNp63α and 14-3-3σ and other 14-3-3 isoforms. *B*, Overexpression of 14-3-3σ had no effect on ΔNp63α levels. PANC-1 cells were transfected with an empty vector (Sham) or with the full-length human 14-3-3σ cDNA that was tagged with HA. *C*, Silencing 14-3-3σ does not affect ΔNp63α. T3M4 cells were infected with lentivirus containing control or 14-3-3σ specific shRNAs (sh 14-3-3σ 1 and 2), whole cell lysates were prepared 72 hrs post infection and probed for ΔNp63α and 14-3-3σ. *D–F*. 14-3-3σ is required for the oncogenic effects of ΔNp63α in PDAC. PANC-1 cells were infected with control sh RNA or sh 14-3-3σ 2. Subsequent to that, cells were transiently transfected with ΔNp63α-expressing vector or vector control. *D*, Anchorage-independent growth. Cells were incubated in soft agar as described in the methods. Data are the mean ± SE of three independent experiments. *E*, Cells (5×10^4^/well) were subjected to a migration assay in Transwell chambers in the presence of EGF (1 nmol/L) for 18 hours in duplicates. Data are the mean ± SE of three independent experiments. *F*, Cells were subjected to invasion in Matrigel chambers in the presence of EGF (1 nmol/L) for 20 h in duplicates. Data are the mean ± SE of three independent experiments.

To determine if ΔNp63α modulates 14-3-3σ expression in pancreatic cancer cells, we introduced ΔNp63α cDNA via adenoviral infection in ASPC-1 and PANC-1 cells, which express low levels of 14-3-3σ and ΔNp63α. We observed an increase in 14-3-3σ protein levels in the ΔNp63α overexpressing cells compared to the control-infected cells (Fig. 5A). This ΔNp63α-mediated increase in 14-3-3σ was not associated with alterations of protein levels of the other 14-3-3 isoforms (Fig. 5A). By contrast, manipulation of 14-3-3σ levels in PANC-1 and T3M4 cells did not have an effect on ΔNp63α (Fig. 5B and 5C).

Next, we determined whether upregulation of 14-3-3σ by ΔNp63α is of biological significance in PDAC. Using a lentiviral based approach we silenced endogenous 14-3-3σ in PANC-1 cells. In such cells, enforced expression of ΔNp63α failed to upregulate 14-3-3σ (data not shown). Consistent with our previous observations, loss of 14-3-3σ did not affect migration abilities of PANC-1 cells in the presence of EGF [[11], Fig. 5E]. Similarly, ΔNp63α-mediated enhancement in cell motility was not influenced by the lack of 14-3-3σ (Fig. 5E). By contrast, 14-3-3σ was required for ΔNp63α-mediated anchorage-independent growth and invasion in presence of EGF (Fig. 5D and 5F). Thus, ΔNp63α upregulates 14-3-3σ in pancreatic cancer cells which then contributes to ΔNp63α-mediated anchorage-independent growth and invasion.

### ΔNp63α is a transcriptional activator of EGFR and 14-3-3σ in pancreatic cancer

ΔNp63α is a regulator of the transcriptional activities of the p53 family member proteins. As such, ΔNp63α may inhibit transcription via its dominant negative effect on p53 and TAp63γ [27]. In addition, the ΔN variant is able to trans-activate p53 target genes via binding to p53 responsive elements within the corresponding gene promoters. While TAp63γ is a potent gene trans-activator due to the absence of an auto-inhibitory domain [27], recent work demonstrated that it has a very low ability to bind the p53 responsive element within the EGFR promoter, and in fact represses promoter activity [28]. Since pancreatic cancer cell lines expressed very low levels of the TAp63 isoform, and since we found no correlation between ΔNp63α and TAp63γ expression in those cell lines (Fig. 1A), the dominant negative effect of ΔNp63α was an unlikely explanation of the changes in EGFR and 14-3-3σ expression. Both EGFR and 14-3-3σ promoters contain well-conserved p53 binding sites, suggesting they are transcriptional targets of ΔNp63α in PDAC. The 14-3-3σ promoter is known to be unmethylated in pancreatic cancer cell lines [29].

**Figure 6:**
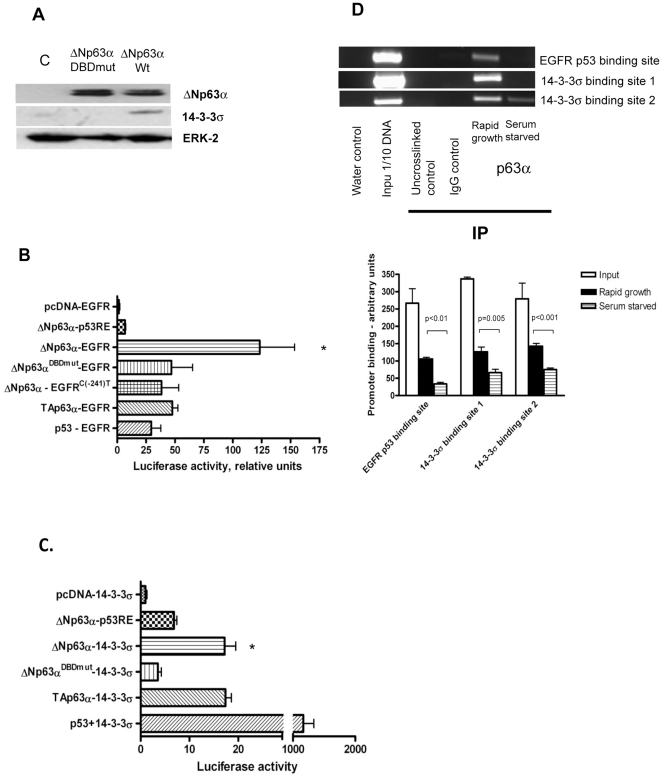
ΔNp63α is a transcriptional activator of EGFR and 14-3-3σ in pancreatic cancer cells. *A*, ΔNp63α^DBDmut^ does not upregulate 14-3-3σ. *B and C*, ΔNp63α upregulates EGFR and 14-3-3σ promoter in a luciferase assay. PANC-1 cells were transfected with wildtype EGFR, mutant EGFR or 14-3-3σ promoter luciferase construct as indicated, along with pCMVβ plasmid, with or without wildtype p53, ΔNp63α, ΔNp63α^DBDmut^, or TAp63α. At 48–72 h after transfection, the luciferase activity was determined. The transfection efficiency was standardized against β-galactosidase activity. Results are indicative of four independent experiments. *, p<0.0001. *D*, p63 binds EGFR and 14-3-3σ promoters in T3M4 cells. Binding is decreased upon serum-starvation. T3M4 cells were grown in medium containing 10% FBS (rapidly growing cells) or in serum-free medium (predominantly resting cells). Cells were pelleted and ChIP was performed as described in materials and methods. Uncrosslinked protein lysate, protein lysate immunoprecipitated with an irrelevant antibody, and a protein lysate immunoprecipitated with p63 antibody, but where irrelevant DNA sequences located ∼3000 bp downstream of the 14-3-3σ or EGFR promoter regions were amplified served as negative controls (the latter not shown).

ΔNp63α requires an intact DNA-binding domain for its transcriptional activity [18]. We generated a mutant vector where we introduced a missense mutation into human ΔNp63α cDNA sequence resulting in a single amino acid substitution at position 202 (ΔNp63α^DBDmut^; Fig. S3). Enforced expression of ΔNp63α^DBDmut^ in PANC-1 cells resulted in a detectable ΔNp63α protein, but failed to upregulate 14-3-3σ (Fig. 6A) indicating that the ability of ΔNp63α to upregulate 14-3-3σ is dependent upon its ability to bind to DNA.

We tested if ΔNp63α was able to activate EGFR promoter in an *in vitro* luciferase assay. The details of the p53 binding sites within the EGFR promoter and the corresponding luciferase vector were published previously [10]. Ectopic expression of ΔNp63α resulted in a dramatic increase of the activity of EGFR reporter plasmid in PANC-1 cells (Fig. 6B). The effect of ΔNp63α significantly exceeded that of wild-type p53 and TAp63α. Next, we introduced a single nucleotide mutation within the p53 BS 1 of the EGFR promoter. Specifically, cytosine was replaced with thymidine at position −241 (EGFR^C(−241)T^). As a further confirmation of our findings, co-transfection of a mutant EGFR reporter plasmid with wild-type ΔNp63α, or wild-type EGFR reporter plasmid and mutant ΔNp63α^DBDmut^ resulted in attenuated luciferase activity (Fig. 6B). Consistent with this observation, ectopic expression of ΔNp63α resulted in a significant increase of the 14-3-3σ reporter plasmid activity in PANC-1 cells, which was attenuated when a ΔNp63α^DBDmut^ expression vector was co-transfected in place of wild type ΔNp63α (Fig. 6C).

Next, we tested whether ΔNp63α is capable of binding EGFR and 14-3-3σ promoters at their corresponding p53 binding sites. Our ChIP experiments revealed that ΔNp63α exhibited significant binding to p53 binding sites in the endogenous EGFR and 14-3-3σ promoters in T3M4 cells (Fig. 6D). Chromatin binding depended on the growth phase of T3M4 cells as serum starvation resulted in a significant decrease in promoter occupancy by ΔNp63α (Fig. 6D), while ΔNp63α protein expression was unchanged (data not shown).

We next sought to determine whether the ability of ΔNp63α to trans-activate EGFR and 14-3-3σ promoters was unique to pancreatic cancer. We studied HEK293 cells, a transformed non-malignant cell line, and H1299 cells, a non-small cell lung cancer cell line [30]. Similar to our pancreatic cancer cells, H1299 cells carry a mutant p53. While ΔNp63α is known to be expressed in non-small cell lung cancer [31], H1299 cells predominantly express the TAp63 variant [30].

**Figure 7:**
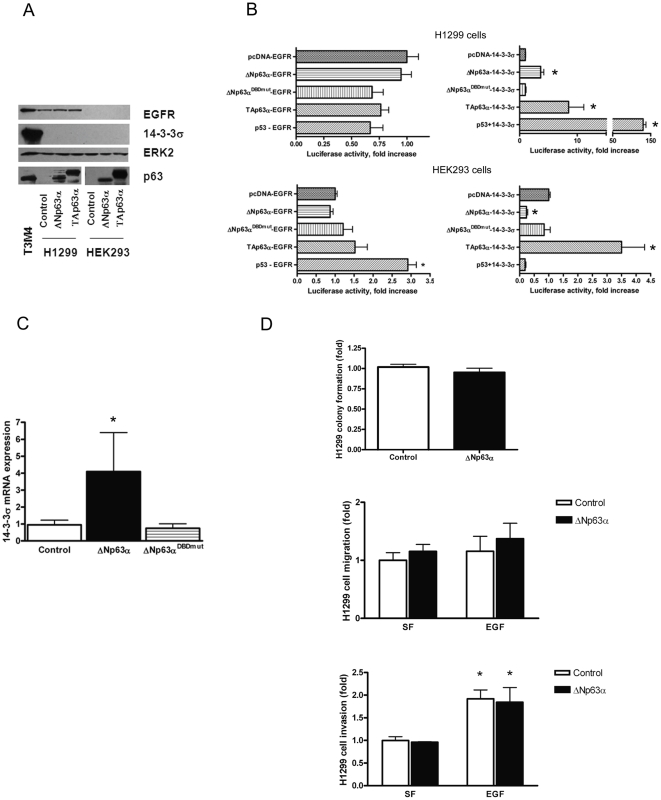
A distinct role of ΔNp63α in HEK293 and H1299 cells. *A*, ΔNp63α does not affect EGFR or 14-3-3σ protein levels in HEK293 or H1299 cells. Representative blot of three independent experiments is shown. *B*, ΔNp63α fails to upregulate EGFR promoter but modulates 14-3-3σ promoter in a luciferase reporter assay. Cells were transfected with wildtype EGFR or 14-3-3σ promoter luciferase construct as indicated, along with pCMVβ plasmid, with or without wildtype p53, ΔNp63α, ΔNp63α^DBDmut^, or TAp63α. At 48–72 h after transfection, the luciferase activity was determined. The transfection efficiency was standardized against β-galactosidase activity. Results are indicative of three independent experiments performed in duplicates. *C*, ΔNp63α increases 14-3-3σ mRNA transcript levels in H1299 cells. H1299 cells were transfected with ΔNp63α, ΔNp63α^DBDmut^ or vector control. At 36 hours total RNA was isolated, reverse-transcribed and subjected to real-time PCR with probe specific for 14-3-3σ. Results were normalized to 18S levels. Data are the mean of two independent experiments done in duplicates in which similar results were obtained. *D*, ΔNp63α does not enhance anchorage-independent growth, migration or invasion in H1299 cells. H1299 cells were transfected with ΔNp63α or vector control. Soft agar, transwell migration and invasion assays were performed as described above. *, p<0.05 compared with control.

Both HEK293 and H1299 cells expressed lower levels of p63 and 14-3-3σ compared to T3M4 pancreatic cancer cells (Fig. 7A). Engineered expression of ΔNp63α in these cells did not lead to an increase in either EGFR or 14-3-3σ protein levels (Fig. 7A). Similarly, ΔNp63α did not activate the EGFR promoter in either cell line in an *in vitro* luciferase reporter assay (Fig. 7B, left panels). By contrast, ΔNp63α transactivated the 14-3-3σ promoter in H1299 cells, albeit to a lesser degree than in pancreatic cancer cells (Fig. 7B, top right panel). In HEK293 cells, ΔNp63α repressed the 14-3-3σ promoter, similar to what was previously reported in human keratinocytes [25], whereas in H1299 cells enforced expression of ΔNp63α led to an increase in 14-3-3σ mRNA transcript levels (Fig. 7C).

ΔNp63 has been shown to enhance proliferative capacity in normal and malignant cells of epithelial origin [32]–[34]. While ΔNp63α stimulated anchorage-independent growth, invasion and migration of pancreatic cancer cells and sensitized them to EGF, such effects were not observed in H1299 cells (Fig. 7D). Collectively, these data indicate that ΔNp63α binds and trans-activates the EGFR and 14-3-3σ promoters in pancreatic cancer cells and that these actions of ΔNp63α do not necessarily occur in other cell types.

## Discussion

Genetic defects which result in inactivation of tumor suppression genes and accumulation of oncogenic alleles promote development and progression of PDAC. p53 is a prototypic tumor suppressor that is vital in cell growth control. However, normal p53 function is commonly lost in PDAC. p53 homolog p63 possesses an N-terminal trans-activation domain (TA), a DNA-binding domain (DBD) and a C-terminal oligomerization domain (OD). Studies in knockout mice demonstrated that despite structural similarity with p53, p63 has distinct functional properties [35]. Moreover, variability of expression of six known p63 isoforms among normal and malignant tissues suggests that the functional role of p63 is dependent on the cellular context. TAp63 isoforms may carry an anti-oncogenic potential as they mediate Ras-induced cellular senescence, antagonize tumorigenesis *in vivo* and suppress development of metastases through regulation of microRNA network [36], [37]. The role of ΔNp63 in cancer is less clear. Recent studies have indicated that ΔNp63α is necessary and sufficient to by-pass oncogene-induced senescence suggesting that it plays a critical role in the very early steps of cancer initiation [38]. Overexpression of ΔNp63 is found in cancers of epithelial origin (lung, head and neck), where it promotes tumor survival [7]. ΔNp63 has been shown to enhance the proliferative capacity of both epithelial stem cells and cancer cells, and loss of p63 reduced the proliferative rate of MCF-7 breast cancer cells [32], [33]. However, the role of p63 in PDAC is not known. Here we report that ΔNp63α is the predominantly expressed p63 isoform in pancreatic cancer cell lines. We demonstrate that ectopic expression of ΔNp63α in PANC-1 cells, which have low p63 mRNA transcript and protein levels, resulted in enhancement of anchorage-independent growth, cell motility and invasion. Conversely, shRNA-mediated suppression of ΔNp63α in T3M4 cells resulted in the opposite effect, i.e. decreased proliferation, pro-migratory and pro-invasive responses. In earlier reports, downregulation of endogenous p63 led to enhanced apoptosis in head and neck squamous cell cancer cell lines irrespective of the p53 gene status [7]. By contrast, exogenous ΔNp63α induced cell cycle arrest and apoptosis in p53-null non-small cell lung cancer cells [39]. In our studies, neither engineered expression of ΔNp63α, nor downregulation of endogenous ΔNp63α induced apoptosis in p53-mutant pancreatic cancer cells (data not shown). While migration and invasion are both paramount to embryonic development and wound healing in normal tissues, in cancer those processes are involved in local tumor invasion and metastasis. Hence, these data underscore the importance of ΔNp63α in tumor progression in PDAC.

Tyrosine kinase and serine/threonine kinase pathways regulate multiple cellular processes and are major effectors in PDAC development [40]. EGFR is a 170 kDa transmembrane glycoprotein of the ErbB family of tyrosine kinase growth factor receptors. EGFR activates Ras/Raf/MAPK-ERK and PI-3 kinase/Akt pathways leading to increased cell proliferation, reduced apoptosis, increased angiogenesis, enhanced motility, invasion and metastasis [41]. EGFR signaling blockade via a dominant negative mechanism leads to reduction in mitogenic activity in pancreatic cancer cells through decreased activation of MAPK pathway [42]. In our experiments, ΔNp63α enhanced EGF-mediated motogenic effects and potentiated EGFR signaling as evidenced by enhanced phosphorylation of ERK and Akt and increased activation of JNK in PANC-1ΔN cells. We and others previously reported that EGF is capable of promoting cancer cell growth through JNK activation [42], [43]. Suppression of ΔNp63α in T3M4 cells resulted in a remarkable attenuation of EGF-mediated invasion. Collectively, these observations suggest that PDAC requires ΔNp63α to fulfill its pro-invasive potential in response to EGF stimulation.

Clinical trials of EGFR-targeting agents reported modest effects on patient survival in PDAC [44], [45]. The therapeutic efficacy of these agents could be improved if regulation of EGFR signaling was better understood. Here we provide evidence of a functional interplay between ΔNp63α and EGFR in pancreatic cancer. Ectopic expression of ΔNp63α, but not TAp63α, resulted in increased EGFR protein levels in PANC-1 cells. Conversely, downregulation of ΔNp63α in T3M4 cells led to a decreased expression of EGFR. It was initially felt that whereas TAp63 is a strong gene trans-activator, ΔNp63 functions as a dominant negative isoform [27]. However, recent findings point out that ΔNp63 is able to trans-activate p53 target genes as well as distinct targets [46], [47]. TAp63γ has been reported to repress EGFR promoter activity in H1299 lung cancer cells [28]. However, we determined that ΔNp63α is a transcriptional activator of EGFR in pancreatic cancer cells, but not in HEK293 or H1299 cells. Moreover, compared to p53, ΔNp63α was a strong trans-activator of EGFR in a functional reporter assay, and ChIP experiments confirmed that ΔNp63α binds the EGFR promoter at the p53-binding site. By contrast, TAp63α was a weak transcriptional activator of EGFR in a functional reporter assay. However, downregulation of TAp63 isoforms also decreased EGFR expression in T3M4 cells, suggesting that in those cells TAp63 isoforms could still be contributing to EGFR expression.

PDAC is notoriously resistant to conventional chemotherapy agents. This is partly explained by frequent inactivation of p53 in pancreatic tumors and thus unresponsiveness to genotoxic stress. Here we demonstrate that ΔNp63α further contributes to chemotherapy resistance in PDAC. While overexpression of ΔNp63α in PANC-1 cells resulted in a marginal increase in apoptotic death upon exposure to cisplatin, its downregulation in T3M4 cells led to a markedly increased sensitivity of those cells to cisplatin-induced apoptosis. Similar to our observations in pancreatic cancer cells, cisplatin induces proteosomal degradation of ΔNp63α in other cell types [24]. Additional mechanisms that contribute to cisplatin resistance in pancreatic cancer include overexpression of cyclin D1 [48] and multidrug resistance-associated proteins [49]. Moreover, ΔNp63α itself may promote chemoresistance through several mechanisms. For example, it can have a dominant negative effect on the proapoptotic partners within the p53 family, as suggested by earlier experiments performed in head and neck and breast cancer [6]. Although ΔNp63α contributed to chemoresistance through regulation of Akt1 expression in ovarian and head and neck cancer [50], we did not observe such effect in pancreatic cancer cells (Fig. 3A). However, we highlight a new potential mechanism of ΔNp63α-mediated chemoresistance in PDAC. In our earlier experiments, 14-3-3σ dramatically increased chemoresistance and enhanced the pro-invasive potential of pancreatic cancer cells [11]. Here we demonstrate that expression of 14-3-3σ protein correlates with ΔNp63α levels in those cells. While others have identified 14-3-3σ as a ΔNp63 repression target in human embryonic keratinocytes [25], we established that ΔNp63α activates the 14-3-3σ promoter and upregulates 14-3-3σ protein expression in pancreatic cancer cells (and in H1299 lung cancer cells), thus decreasing sensitivity of pancreatic cancer cells to cisplatin-induced apoptosis. This discrepancy provides additional evidence that ΔNp63α actions are tissue-specific, and its function in cancer is distinct from its role in embryonic cells. We also found that 14-3-3σ promoter binding by ΔNp63 was more pronounced in rapidly cycling as compared with resting T3M4 cells. This suggests that at least some ΔNp63α target sites may be poorly accessible in quiescent cells and require chromatin remodeling to occur prior to binding, further implicating cellular context in modulation of p63 function.

In our immunoprecipitation experiments we found no interaction between p63 and 14-3-3σ proteins (data not shown), however 14-3-3σ contributed to the mitogenic and motogenic effects of ΔNp63α in PDAC. Lentiviral knockdown of 14-3-3σ resulted in a dramatic attenuation of the effects of ΔNp63α on anchorage-independent growth and EGF-stimulated invasion of PANC-1 cells. Silencing of 14-3-3σ had no consequences on the effects of ΔNp63α on cell motility, leading to the conclusion that ΔNp63α affects migration through potentiation of the other signaling pathways, i.e. EGFR and integrins.

p63 controls the adhesion program in MCF-10A mammary epithelial tissues through transcriptional regulation of integrins [15]. A number of integrin subunits have been shown to be upregulated in PDAC [51]. We demonstrate that ΔNp63α modulates expression of β1-integrins, which are involved in regulation of cell adhesion and migration of tumor on stroma proteins, thus further implicating ΔNp63α in pancreatic carcinogenesis. Importantly, integrin and EGFR pathways are closely intertwined and orchestrate cancer growth and invasion. Integrin-mediated adhesion of cells to extracellular matrix induces EGFR activation in a ligand-independent manner, while EGFR regulates integrin signaling and is necessary for adhesion-induced activation of ERK and other signaling molecules [52]. Cross-talk between integrins and EGFR family members affects multiple aspects of tumor progression, including proliferation, migration and invasion [53]. For example, blockade of integrin α_v_β_5_ reverses the EGF-stimulated invasion and metastasis in pancreatic cancer cells [54]. On the other hand, overexpression of β1-integrin has been shown to correlate with acquired resistance to EGFR inhibitors in lung cancer [55]. The unique ability of ΔNp63α to regulate both EGFR and β1-integrin in PDAC renders it a particularly promising therapeutic target.

In conclusion, ΔNp63α is the predominantly expressed p63 variant in PDAC cell lines with oncogenic properties. In PANC-1 and T3M4 cells ΔNp63α enhanced anchorage independent growth, cell proliferation, and basal and EGF-stimulated motility and invasion and conferred chemoresistance. The oncogenic effects of ΔNp63α were mediated via transcriptional activation of EGFR and 14-3-3σ. Our observations indicate that targeting ΔNp63α and 14-3-3σ may present a strategy to potentiate the efficacy of EGFR-targeting therapies in PDAC.

## Supporting Information

Table S1 **p63 primer sequence and expected size of PCR products.**
(DOC)

Figure S1 **Genomic structure of human p63 and location of primers and sh RNA complementary sites.** Genomic structure of human p63 on chromosome 3 and schematic representation of the six variants of p63. TA – trans-activation domain, DBD – DNA-binding domain, OD – oligomerization domain, SAM – sterile alpha motif, TID – transactivational inhibitory domain.
(TIF)

Figure S2 **Erlotinib attenuates ΔNp63α-mediated enhancement of migration, invasion and anchorage-independent growth in PANC-1 cells.** *A*, Erlotinib attenuates ΔNp63α-mediated anchorage-independent growth of PANC-1 cells in soft agar assay. Soft agar assay was performed as described previously in presence of 1 µM erlotinib or vehicle control. *B*, PANC-1 cells (5×10^4^/well) were subjected to a migration assay in Transwell chambers in presence of EGF (1 nmol/L) as described above, with or without 1 µM erlotinib. *C*, PANC-1 cells (1×10^4^/well) were subjected to invasion assay in Matrigel chambers in presence of EGF (1 nmol/L) as described above, with or without 1 µM erlotinib. *, p<0.05 compared with control.
(TIF)

Figure S3 **ΔNp63α^DBDmut^ cDNA-expressing vector. Partial sequence of the ΔNp63α cDNA shown (nucleotides 601 to 612, within DBD).** Substituted nucleotide is shown in capital.
(TIF)
