# Correction: *DeltaNp63alpha*-Mediated Induction of Epidermal Growth Factor Receptor Promotes Pancreatic Cancer Cell Growth and Chemoresistance

**DOI:** 10.1371/journal.pone.0192927

**Published:** 2018-02-08

**Authors:** Alexey V. Danilov, Divas Neupane, Archana Sidalaghatta Nagaraja, Elena V. Feofanova, Leigh Ann Humphries, James DiRenzo, Murray Korc

There is an error in Fig 2. Fig 2D includes an incorrect image among the representative images showing cell migration. The image for SF (0h) for ΔNp63α is a duplication of the image for EGF (0h) for Control.

**Fig 2:**
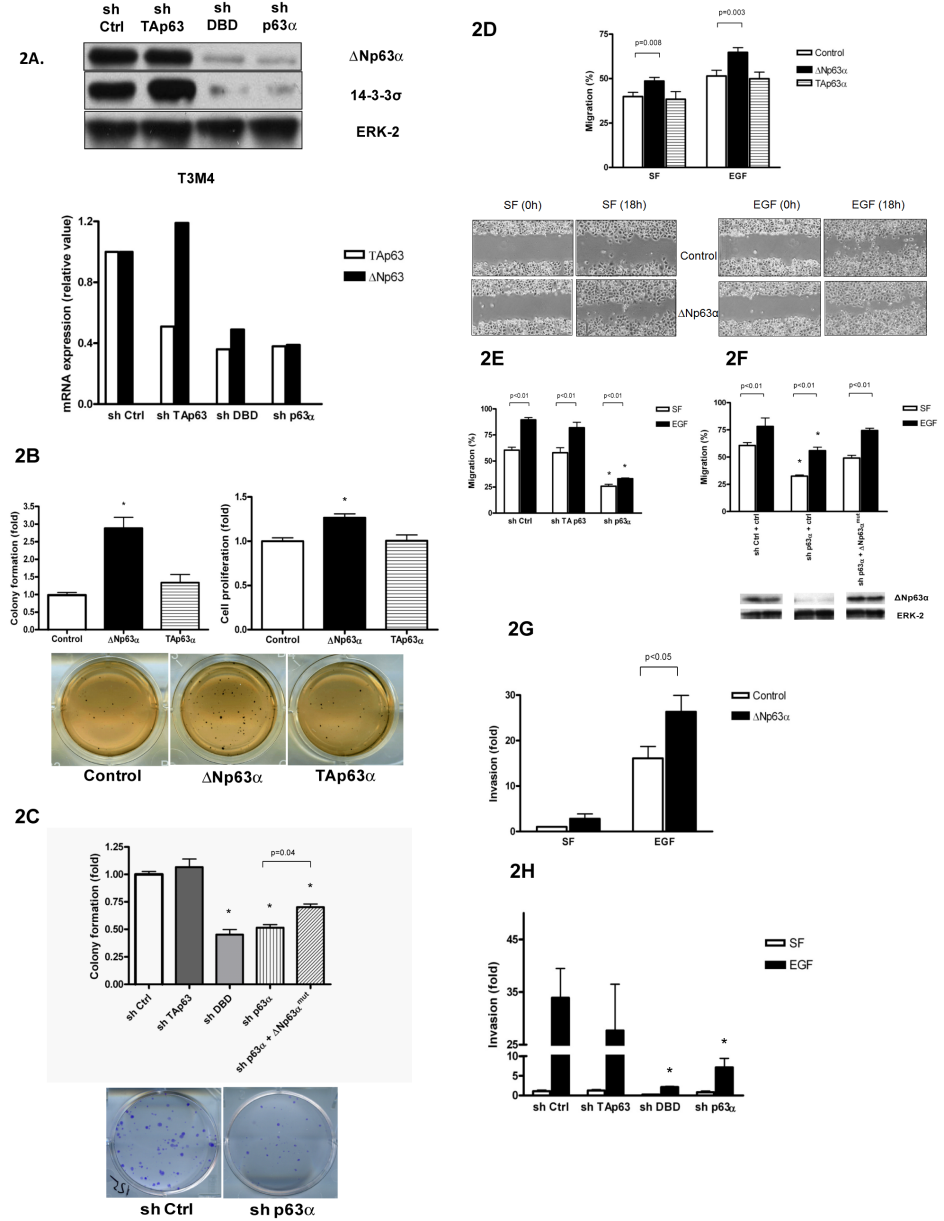
Effect of ΔNp63α on anchorage-independent growth, motility, and invasion in pancreatic cancer cells. *A*, T3M4 cells were infected with GFP-expressing virus, or p63-specific shRNA complementary to DBD, TA and α-specific domains of p63. Whole-cell protein lysates were subjected to immunoblotting (top panel). Total RNA was isolated, reverse-transcribed and subjected to real-time PCR with probes specific for ΔN and TA isoforms of p63. Results were normalized to 18S levels (bottom panel). Knockdown of p63 isoforms was routinely monitored during the subsequent experiments. *B*, ΔNp63α stimulates anchorage-independent growth of PANC-1 cells in soft agar assay (left and bottom panels) and cell proliferation in MTT assay (right panel). PANC-1 cells were transiently transfected with ΔNp63α-expressing vector, TAp63α or control vector. Cell were plated in soft agar at a density of 1500/well of a 12-well plate, four wells per sample. Colonies were counted after 14 days of incubation. For MTT assay cells were plated in 96-well plates, six wells per sample. MTT was added after incubation for 48 h. Data are the mean ± SE of four independent experiments. *, p<0.001 compared with control. *C*, Downregulation of ΔNp63α slows proliferation of T3M4 cells in a clonogenic assay. Reconstitution of ΔNp63α partially restores the proliferative ability. Cells were plated on 6 well plates at a density of 500 cells/well. 14 days later, plates were fixed in 3:1 methanol:glacial acetic acid and stained with 2% crystal violet. Data are the mean ± SE of three independent experiments done in triplicates. *, p<0.01 compared with control (sh ctrl). *D–F*, Effect of p63 on cell motility measured in wound-healing assays in PANC-1 (D) and T3M4 cells (*E*, *F*). Cells were incubated in serum-free (SF) conditions in the absence or presence of 1 nM EGF for 18 h after making a scratch. Quantitative analysis of the images was performed. Ectopic expression of mouse ΔNp63α in sh p63α T3M4 cells resulted in a partial restoration of cell motility (F); corresponding ΔNp63α protein levels shown below. Data are the m ± SE of at least three independent experiments. Representative pictures shown (magnification ×40). *, p<0.001 compared with control (sh ctrl). *G and H*, Effect of ΔNp63α on the invasion in Matrigel chambers. PANC-1 (*G*) or T3M4 (H) cells were plated in Matrigel chambers (5×104/ml) and incubated in absence (SF) or presence of 1 nM EGF for 18 hours. Effect was normalized to invasion of control in SF conditions. *, p<0.001 compared with control (sh ctrl).

Please see the corrected Fig 2 here.
