# Supplementary material for: DeltaNp63alpha-Mediated Induction of Epidermal Growth Factor Receptor Promotes Pancreatic Cancer Cell Growth and Chemoresistance
Source: PLoS One. 2011 Oct 28;6(10):e26815. doi: 10.1371/journal.pone.0026815 (PMC3203907; doi:10.1371/journal.pone.0026815)
Supplement: Table S1 — p63 primer sequence and expected size of PCR products. (DOC) [file pone.0026815.s001.doc]

| **Forward primer** | **Reverse** | **Product length (bp)** | **Gene specificity** |
| --- | --- | --- | --- |
| I  TTAGCATGGACTGTATCCGC | III  ACTTGCCAGATCATCCATGG | 1486 | *TAp63α* |
|  | IV  TCAGACTTGCCAGATCCTG | 1396  1490 | *TAp63β*  *TAp63α* |
|  | V  AAGCTCATTCCTGAAGCAGG | 1108 | *TAp63γ* |
| II  CCAGACTCAATTTAGTGAGC | III  ACTTGCCAGATCATCCATGG | 1452 | *∆Np63α* |
|  | IV  TCAGACTTGCCAGATCCTG | 1362  1456 | *∆Np63β*  *∆Np63α* |
|  | V  AAGCTCATTCCTGAAGCAGG | 1074 | *∆Np63γ* |
