# Supplementary figures and images for: DeltaNp63alpha-Mediated Induction of Epidermal Growth Factor Receptor Promotes Pancreatic Cancer Cell Growth and Chemoresistance
Source: PLoS One. 2011 Oct 28;6(10):e26815. doi: 10.1371/journal.pone.0026815 (PMC3203907; doi:10.1371/journal.pone.0026815)

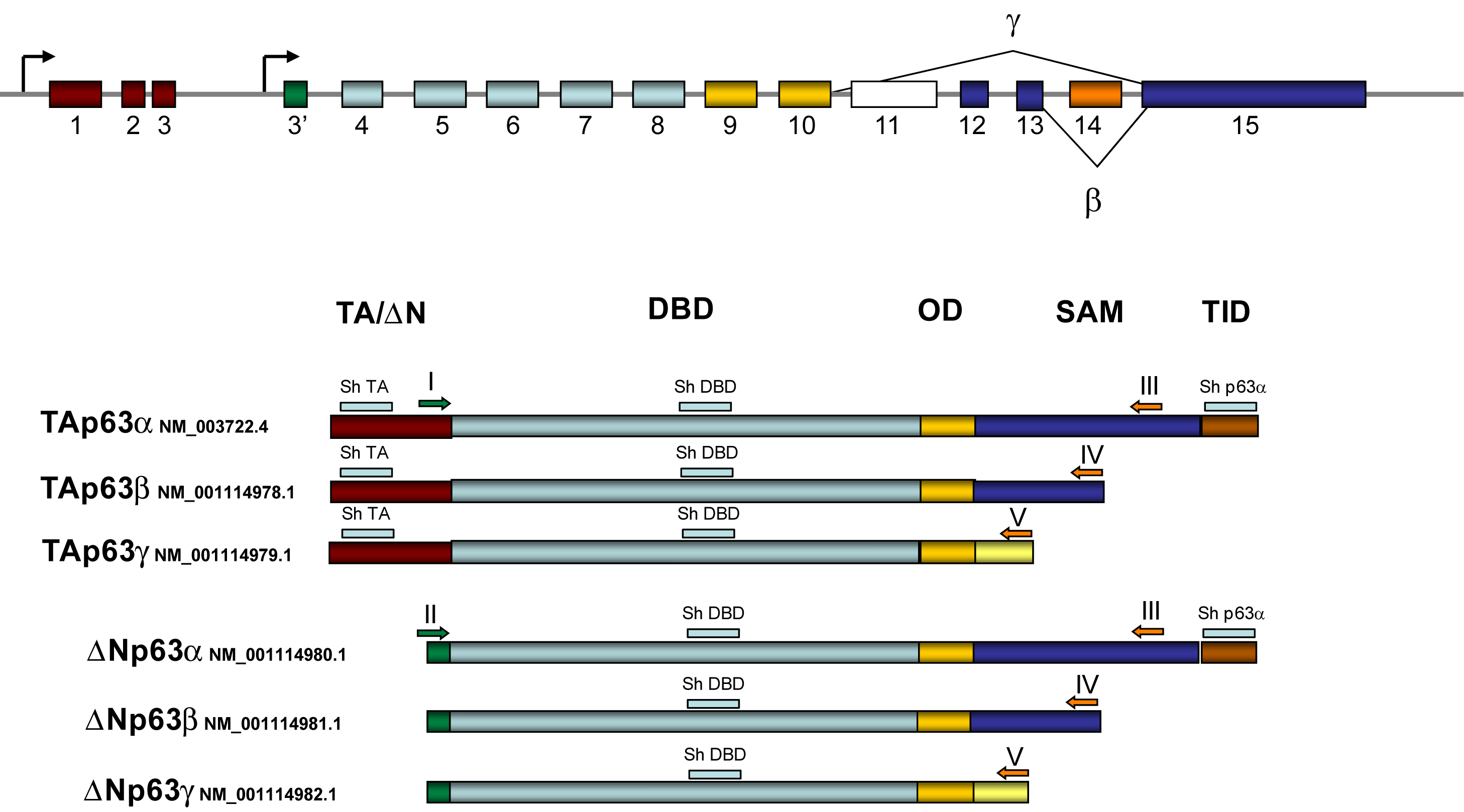

Supplement: Figure S1 — Genomic structure of human p63 and location of primers and sh RNA complementary sites. Genomic structure of human p63 on chromosome 3 and schematic representation of the six variants of p63. TA – trans-activation domain, DBD – DNA-binding domain, OD – oligomerization domain, SAM – sterile alpha motif, TID – transactivational inhibitory domain. (TIF) [file pone.0026815.s002.tif]

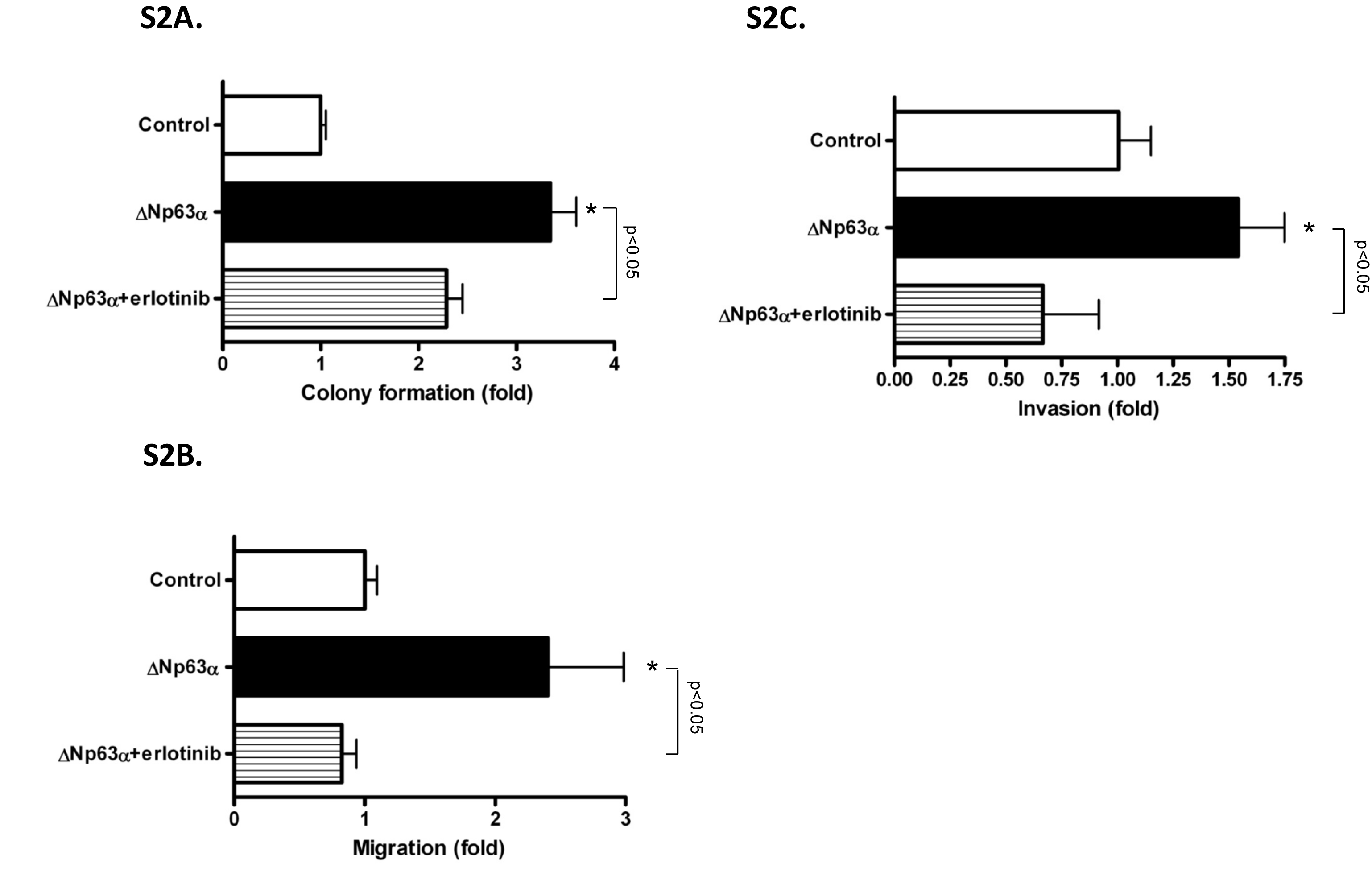

Supplement: Figure S2 — Erlotinib attenuates ΔNp63α-mediated enhancement of migration, invasion and anchorage-independent growth in PANC-1 cells. A, Erlotinib attenuates ΔNp63α-mediated anchorage-independent growth of PANC-1 cells in soft agar assay. Soft agar assay was performed as described previously in presence of 1 µM erlotinib or vehicle control. B, PANC-1 cells (5×10^4^/well) were subjected to a migration assay in Transwell chambers in presence of EGF (1 nmol/L) as described above, with or without 1 µM erlotinib. C, PANC-1 cells (1×10^4^/well) were subjected to invasion assay in Matrigel chambers in presence of EGF (1 nmol/L) as described above, with or without 1 µM erlotinib. *, p<0.05 compared with control. (TIF) [file pone.0026815.s003.tif]

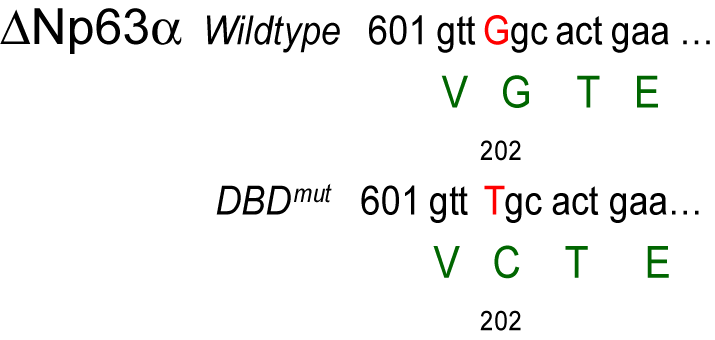

Supplement: Figure S3 — ΔNp63αDBDmut cDNA-expressing vector. Partial sequence of the ΔNp63α cDNA shown (nucleotides 601 to 612, within DBD). Substituted nucleotide is shown in capital. (TIF) [file pone.0026815.s004.tif]
